# Case of Intermittent Fever Complicated with Purpura Hæmorrhagica: Cured by Quinine and Nitric Acid

**Published:** 1846-02

**Authors:** W. W. Leak

**Affiliations:** Cass county, Ga.


### SELECTIONS

FROM

### AMERICAN AND FOREIGN JOURNALS.

---

*Case of Intermittent Fever complicated with Purpura Hæmorrhagica: cured by Quinine and Nitric Acid.* By W. W. Leak, M.D., of Cass county, Ga.—Mr. ——, aged 75 years, a wagon-maker by trade, of plethoric habit and somewhat intemperate—says he has been unwell for two weeks, and on the 29th of Sept. had a chill followed by fever. Soon after this there was an appearance of blotches over various parts of the body, especially on the lower extremities; he had also some spitting of blood; and on the 30th another chill and fever, with vomiting of blood.

At 3 o'clock, P.M., of the 30th, he presented the following symptoms:—countenance pale; headache; pulse a little more feeble than natural, and soft; has cough; some oppression in the chest, and expectorates a little blood; oozing of blood from the mouth and nose; has no appetite; some tenderness over epigastric region; extravasation of blood under the cuticle on various parts of the body, especially on the lower extremities and neck; some bloody vesicles on the tongue as large as peas.

*Prescription.*—V. S. 4 to 6 ounces, which relieved the pain in head and chest.

℞ Calomel, 6 grs.
Dover's powder, 6 grs.
Acetate of lead, 3 grs.
Mix.

Divide into three powders, and give one every third hour. An ounce of castor oil to be taken at night; warm pediluvium with salt. Eight grs. sulph. of quinine, 2 grs. to be given every hour, commencing at 7 o'clock, A.M.

Oct. 1st, 8, A.M. Patient better in all respects, except there is no disappearance of the blotches; has had no cough since the first powder was taken.

*Prescription.*—10 grs. of calomel, to be followed by oil; a teaspoonful of elixir vitriol to one pint of sage tea, to be taken during the day; acetate of lead and Dover's powder to be given if the hæmorrhage returned; 20 grs. sulph. quinine, one-fourth to be given each hour, commencing in the morning at 7 o'clock.

2d, 8, A.M. Had a chill yesterday at 1 o'clock, followed by hæmorrhage from the mucous surfaces. Pulse 110, and quite feeble. In consultation with my friend, Dr. Milner, we determined on giving the nitric acid according to the formula in the August No. of the Southern Medical and Surgical Journal, of Dr. J. J. Bradford.

℞. Nitric acid, ʒi.

Pulv. gum arabic.

White sugar, *āā.* ʒii.

Water, ℥v.

Mix.

Of this, we gave one tablespoonful every six hours, instead of a half tablespoonful, as is recommended by Dr. B.

3, P.M Patient is easy; pulse 100; hæmorrhage less. To continue the acid, with 25 grains sulphate of quinine, onefonrth to be given each hour, commencing in the morning at 7 o'clock.

3d, 8, A.M. Patient rested well last night; had no hæmorrhage except per rectum and urethram, which was not so copious as previously. Pulse 80. Continue the acid.

5, P.M Had no chill to-day. Pulse 72; complains of his head and ears ringing; the blotches are disappearing; his bowels have been moved once or twice. 12 grains quinine to be given in the morning in 3 gr. doses, commencing at 7 o'clock.

4th. Improvement in all respects; blotches disappearing very fast. The acid to be continued for a few days. Dismissed the case to day.

Although this patient has had two or three chills since, we learn there has been no recurrence of the purpura.

The indication in this case was to check the chill, and though the disease complicating it, purpura hæmorrhagica, was abated by the prescriptions first employed, still we think its cure may be clearly attributed to the nitric acid.—*Southern Medical and Surgical Journal.*

